# Does survey mode matter? Comparing in-person and phone agricultural surveys in India

**DOI:** 10.1016/j.jdeveco.2023.103199

**Published:** 2024-01

**Authors:** Ellen Anderson, Travis J. Lybbert, Ashish Shenoy, Rupika Singh, Daniel Stein

**Affiliations:** a University of California, Davis, United States of America; b India Climate Collaborative, India; c IDinsight, United States of America

**Keywords:** Data collection, Phone survey, Survey mode, Agriculture, Measurement error

## Abstract

Ubiquitous mobile phone ownership makes phone surveying an attractive method of low-cost data collection. We explore differences between in-person and phone survey measures of agricultural production collected for an impact evaluation in India. Phone responses have greater mean and variance, a difference that persists even within a subset of respondents that answered the same question over both modes. Treatment effect estimation remains stable across survey mode, but estimates are less precise when using phone data. These patterns are informative for cost and sample size considerations in study design and for aggregating evidence across study sites or time periods.

## Highlights

• We report phone and in-person survey responses on production from Indian farmers.
• Self-reported production has greater mean and variance by phone than in person.
• The difference appears even when the same respondent answered both surveys.
• In an associated impact evaluation, treatment effects are stable across survey mode.
• The phone survey would need to be up to 10.7x larger to achieve the same precision.

## 1 Introduction

Household surveys are standard in economics research, especially in developing economies where administrative records and official statistics are likely to be incomplete due to high degrees of informality (see Deaton, 2005). Traditional survey methods rely on face-to-face interviews with study participants, but the worldwide penetration of information and communication technology makes remote data collection increasingly accessible. In particular, commoditization of mobile phones – an estimated 73% of adults globally and 58%–61% in developing countries now own a mobile phone (ITU, 2022) – enables connectivity in even the most isolated parts of the world.

In this paper, we investigate differences between in-person and phone survey data collected during an agricultural extension experiment in Bihar, India. Phone surveying presents an appealing alternative to in-person data collection because of potential cost savings. Interviewing study participants by phone mitigates the logistical difficulty of physically locating a desired respondent and minimizes enumerator transportation and lodging. However, phone contact can introduce new forms of attrition, and respondents may behave differently when not physically present with an interlocutor. Therefore, it is valuable to explore precisely how to interpret phone responses in relation to comparable in-person data.

Our study leverages data from two parallel impact evaluations of the same underlying program. Evaluators asked a harmonized set of questions on agricultural production, with one team going door-to-door and the other calling by phone. Both surveys independently drew from the same sampling frame, and 42% of households participated in both surveys. We analyze a combined database of responses to the same questions asked to households sampled from the same population that vary only by the mode in which the respondent was contacted. The sampling methodology allows us the quantify both the total difference across survey modes net of mode-specific attrition as well as the pure survey mode effect within households that answered the same question twice.

We conduct two types of comparisons between survey modes. First, we quantify differences in the distribution of self-reported agricultural production for the four most common crop varieties. Phone respondents report 14%–68% more production on average, depending on the crop, and there is greater variance among phone responses for three out of four crops. This pattern is consistent across the output distribution, with larger fractions of phone respondents reporting positive production for three out of four crops and higher production values over the phone at the median, 75th, and 90th percentiles for all four crops.

These differences persist even after accounting for selective attrition by survey mode. Phone respondents in our study tend to be wealthier and more educated on average, mirroring general patterns of mobile phone ownership and use. Nevertheless, the gap between modes at each production decile remains nearly as large when restricting to the subset of households or respondents that participated in both surveys. Within-household and even within-person differences in self-reported production by survey mode explain more than sixty percent of the total measured gap for three out of four crops. There is little evidence that production values were influenced by differential engagement among phone respondents. 87% of participants rounded their response to the nearest five kilograms, and 69% to the nearest ten, but these fractions are nearly identical across survey modes. Therefore, we reject that differences in self-reported production were induced by respondents more carelessly rounding small quantities up over the phone or down in person. We also rule out any systematic bias caused by differences in survey timing.

Second, we compare experimental treatment effects estimated using each method of data collection. Unlike sample means and variances, the within-sample relationship between treatment status and self-reported production remains stable across survey modes. Regression coefficients are similar in magnitude, and we fail to reject equality for any major crop variety. However, we report greater estimation error when using the phone survey data, consistent with higher variance in phone responses.

Taken together, these results can inform research design and evidence aggregation. We show that heterogeneity in the method of contact may introduce bias into comparisons of survey outcomes across populations. Such bias can undermine conclusions about differences between study populations or about the evolution of outcomes within a population over time, such as in subsequent rounds of a panel or repeated cross-sectional survey. To make such comparisons viable, it is necessary to establish reliable indicators that link data across survey modes. We find this issue to be less of a concern for program evaluation.

Our findings also highlight a tradeoff in the use of phone surveying for program evaluation. While it may be cheaper to conduct surveys by phone than in person, the resulting data may be noisier. In such cases, phone-based data collection necessitates larger samples to achieve the same power, offsetting some of the cost savings. In our context there is substantial heterogeneity in the breakeven point: depending on the crop, the phone sample would have needed to be 1.2–10.7 times larger than the in-person sample to estimate treatment effects with the same precision. In general, it would be prudent for researchers to consider noise specific to survey method when calculating power.

Evidence on how survey mode affects data reliability most commonly focuses on self-reported health indicators. Investigators report mixed results on the correspondence between in-person and phone responses, and those showing statistical differences draw no systematic conclusions about types of indicators subject to mode effects or direction of bias (Greenfield et al., 2000, Biemer, 2001, Scherpenzeel and Eichenberger, 2001, St-Pierre and Béland, 2004, Nord and Hopwood, 2007, Ferreira et al., 2011, Mahfoud et al., 2015, Greenleaf et al., 2020). Other comparisons include phone-based measures of consumer valuation (Maguire, 2009, Szolnoki and Hoffmann, 2013), microenterprise data (Garlick et al., 2020), and school performance (Crawfurd et al., 2021). In developing-country agriculture, Kilic et al. (2021) uncover a similar pattern to ours of greater self-reported production by phone than in person among tuber farmers in Malawi.^1^

Our analysis extends this literature in three ways. First, the overlapping sample of respondents allows for within-household estimation of survey mode effects. Only Mahfoud et al. (2015) include this feature, but prime for consistency by advertising phone contact as a check on prior in-person responses.^2^ Second, while most existing work tests for bias in sample means, we also report differences in precision and at various production quantiles. In particular, our finding of greater variance in phone-based data, consistent with a recent study of microenterprises (Garlick et al., 2020), can inform sample size calculations in research design. Third, we investigate how the mode used for data collection affects program evaluation in agriculture. Crawfurd et al. (2021) reach a similar conclusion that survey mode affects measurement of student test scores on average, but does not bias evaluation of an educational intervention.

Research interpreting phone survey data is especially timely following COVID-19 disruptions that forced remote data collection. To accurately quantify the evolution of economic outcomes through the pandemic and beyond, researchers must find ways to relate outcomes across surveys (e.g., Egger et al., 2021, Josephson et al., 2021, Barker et al., 2023 for successful examples). To the extent that lessons learned from the large-scale use of remote data collection during the pandemic (Gourlay et al., 2021, Zezza et al., 2022) enable these practices to remain in place in the future, it will be important to develop methods to establish comparability between pre- and post-pandemic surveys.

Our investigation also relates to the growing body of work on how to aggregate evidence across studies. Many policy evaluations take place in idiosyncratic contexts, and organizations such as 3ie^3^ and Cochrane Reviews^4^ devote substantial resources to drawing general conclusions about policy impacts. Meager (2019) provides an empirical framework for evidence aggregation that disentangles average policy impacts, context-specific heterogeneity, and sampling variation; and Pritchett and Sandefur (2015) argue heterogeneity across contexts can threaten external validity moreso than poor identification. In this paper we demonstrate how and when the mode of survey can introduce study-specific heterogeneity in measured outcomes that is largely uninformative for policy decisions.

## 2 Data and methodology

Data for this study come from two overlapping randomized evaluations of an agricultural extension program to promote pulse cultivation in Bihar, India. The program consisted of offering farmers subsidized inputs to accelerate adoption combined with high-intensity extension to teach best practices through learning-by-doing over a period of two years. In this paper we analyze data on pulse production collected in the first-year endline, the only round involving both phone and in-person data collection.

The initial intervention began in May 2017, followed by a pre-harvest midline survey conducted in person in December 2017. The 2346 midline respondents, selected at random from the 6971 evaluation households, comprise the sampling frame for the current study. At midline, all sample households reported on demographic characteristics and pre-harvest farm area devoted to pulses. Of these, 1100 were randomly selected for an extended survey with greater detail on socioeconomic status, and this random subset constitutes the endline in-person sample. At midline, 1525 households reported positive pulse area, and this non-random subset constitutes the phone sample. Notably, 711 households were included in both samples. Appendix A provides a full breakdown of sampling assignment and response rates by survey mode.

We report results on household pulse production from first-year endline surveys conducted post-harvest in May–June 2018. We analyze production of the four most common varieties of pulses—pigeon peas (*arhar*), grown by 660 households; red lentils (*masoor*), grown by 854 households; green peas (*mattar*), grown by 398 households; and fava beans (*bakla*), grown by 390 households. Among these, pigeon peas and red lentils were explicitly targeted by extension efforts in the year of study. Fewer than 100 households reported growing any other variety.

Endline data was collected by parallel in-person and phone surveys that asked nearly identical questions about household production by pulse variety conditional on having positive area planted at midline. The two data collection exercises were motivated by a desire to optimize for different research objectives. The phone survey allowed a larger sample size with the hope of generating more power for the primary outcome of pulse production. The in-person survey contained more modules, allowing detailed exploration of secondary outcomes.

In-person surveying was part of a long-term impact evaluation by researchers at the University of California, Davis. Researchers attempted to reach all 1100 extended midline survey respondents. 1055 households answered the survey, corresponding to an in-person attrition rate of 4.1%. Those that had reported positive area devoted to pulses at midline were asked about their production by variety at endline, and in-person surveys included a number of other questions on agricultural production and food consumption. Full evaluation results from the in-person survey are reported by Lybbert et al. (2023).

Phone surveying was used for a short-term cost-effectiveness analysis by researchers at IDinsight. Researchers attempted to reach all 1525 midline households that had reported positive area devoted to pulses. 1266 responded corresponding to an attrition rate of 17.0% by phone. In our study sample, phone ownership is nearly universal so attrition signals either not answering the call or declining to participate. Phone respondents were asked only about pulses production due to time constraints imposed by the survey format. Full evaluation results from the phone survey are reported by Anderson et al. (2022).

To the extent possible, questions about pulse production were identical across surveys. The exact wording is provided in Appendix A. Enumerators in both surveys were instructed to speak to the primary farmer in the household, who had previously been identified in the midline survey. This individual was the respondent in 84% of in-person and 81% of phone surveys. We interpret differences in the difficulty of reaching the desired respondent to be an inherent feature of data collection, and therefore treat it as one channel through which survey mode effects may operate. While both surveys were administered in parallel, the same household was typically not contacted by both modes on the same day. On average, the in-person survey was conducted 7 days after the phone survey, but differences range from 13 days earlier to 26 days later. In Appendix A we verify responses are not systematically related to this variation in timing. The upper tail of all production responses are Winsorized to the 95% level independently by crop and by mode to match how data would have been treated had either survey been conducted in isolation.

This study presents two types of comparisons between in-person and phone survey responses. First, we compare moments in the distribution of self-reported production volume across survey mode. We report the mean, variance, and value at each decile for the four most common pulse varieties, restricting to households that reported positive area planted at midline and were therefore eligible for both surveys. This comparison reveals how inferences about population outcomes differ by survey mode inclusive of any bias introduced by differential attrition by survey respondents.

We next decompose differences in distribution into selection and mode effects. This analysis leverages the fact that 711 households were contacted for both in-person and phone surveying. Out of these, 584 responded to both modes of contact, and in 429 cases the exact same individual answered each time. Variation in self-reported production volume within this overlapping sample can be attributed purely to survey mode, and the characteristics of non-respondents provide evidence about differential attrition bias. We also explore respondent engagement using evidence of rounding to the nearest five or ten.

Second, we investigate how survey mode affects program evaluation. Here we estimate the intention-to-treat (ITT) effect on pulse production separately within each survey, represented by β in

(1) $$Y_{i}=\beta T_{i}+X_{i}\delta +\gamma_{b\left(i\right)}+\epsilon_{i}$$

where $Y_{i}$ represents production for household i living in block (sub-district) b(i), $T_{i}$ is a dummy indicating treatment status, $X_{i}$ is a vector of household controls, and $\gamma_{b\left(i\right)}$ are block-level fixed effects. The coefficient of interest β corresponds to the effect of treatment, and standard errors are clustered at the village level.

This analysis no longer conditions on positive area planted at midline because planting is an endogenous outcome of treatment. Production volume is given by survey response for households with positive area planted and assumed to be zero (though not explicitly asked) for households that previously reported zero area planted.

## 3 Distribution of self-reported outcomes

In this section we analyze differences in self-reported production by survey mode. This analysis is informative for comparisons made across data sets generated using different methods, for example when making inferences about how outcomes evolve over time from different rounds of a panel survey.

**Fig. 1:**
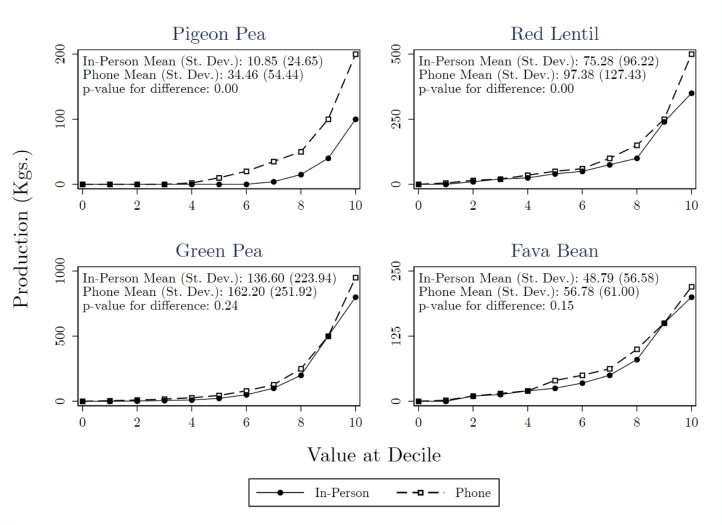
Deciles of production quantity by survey mode. Notes: Self-reported production volume at each decile by crop and by survey mode. Data for each crop includes only those who reported positive area for that crop at midline, and were therefore asked about production of that crop at endline. Top production values are Winsorized to the 95th percentile independently by crop and by mode before computing mean.

The distribution of responses by survey mode are presented in Fig. 1. Each panel plots the value at each decile for the four most common pulse varieties. The solid line represents in-person responses, and the dotted line represents phone responses. Means and standard deviations are also reported for each crop and survey mode.

Data in Fig. 1 restrict to study participants that reported positive area planted at midline, and were therefore asked about production at endline. Nevertheless, some respondents indicate zero harvest production. This is because unfavorable weather conditions in the study year damaged pulse crops, especially pigeon peas. As a result, many households that planted pulses had abandoned cultivation by harvest time.

Results reveal greater self-reported production over the phone than in person. On average, responses range from 14% smaller in person for fava beans up to 68% smaller for pigeon peas. The difference in means is statistically significant at the 1% level for pigeon peas and red lentils, the two crops targeted by the extension program. For all crops except fava beans, there is greater variance in responses over the phone as well.

The pattern of greater production reported in phone surveys appears all along the distribution of responses. A larger fraction of respondents claim non-zero production for all crops, and a chi-squared test rejects equality between survey modes at the 1% level for all but fava bean. Moreover, self-reported production is higher at the median, 75th percentile, and 90th percentile for all four crops. Differences in pigeon pea responses are significant at the 1% level at the median, 75th, and 90th percentiles. Red lentil differences are also significant at the 5% level at the median and 75th percentile, green pea differences are significant at the 10% level at the median, and fava bean differences are significant at the 10% level at the median and 75th percentile. Exact values and test statistics are reported in Appendix B. The consistency of these results indicates that the greater mean and variance of phone responses is not just driven by an exaggerated right tail. As a corollary, we would not be able to reconcile survey modes with a simple fix such as more aggressive winsorization of phone data.

### 3.1 Selective attrition and survey mode effects

We first explore differential attrition as a source of difference by survey mode. Table 1 presents household midline characteristics of the 1525 households that enumerators attempted to contact by phone, which constitute the portion of the sampling frame common to both modes. Column 1 reports means and standard deviations among all households in this population. 711 of these were randomly selected for in-person surveying out of which 698 responded, described in Column 2. Column 3 describes the 1266 households that responded to the phone survey. Columns 4 and 5 report the in-person and phone sample deviations from the sampling frame, respectively. The top panel reports outcomes asked of all study participants, and the bottom panel reports responses from the extended midline subsample.

Attrition was low in person, and endline respondents closely resemble the sampling frame. The only statistically significant deviation is in caste distribution, where there is a slightly lower sampled fraction belonging to a Scheduled Caste or Tribe, almost fully accounted for by Other Backward Castes. All other deviations are quantitatively small and statistically insignificant, consistent with random sampling variation. By contrast, phone survey respondents appear to be selected along typical dimensions. Households in the phone sample are more educated, with heads four percentage points more likely to have completed primary and secondary school, and appear to be wealthier across a range of measures. Phone respondents are less likely to engage in sharecropping, own more assets, are more likely to live in a permanent housing structure, and are less likely to use government assistance such as workfare (MNREGA) or food aid (PDS). These differences in wealth and education are consistent with selection bias commonly observed in phone surveys (see Ambel et al., 2021, Zezza et al., 2022 and citations within).

**Table 1:** Household characteristics by survey response status .

	Pulse growers	Survey respondents		Difference from (1)	
	Sampling frame	In-Person	Phone	In-Person	Phone
	(1)	(2)	(3)	(4)	(5)
Variables from full sample:					
HH Head Age	49.141	49.676	49.172	0.535	0.031
	(15.539)	(15.745)	(15.421)	(0.427)	(0.180)
Caste SC/ST	0.167	0.126	0.165	−0.041⁎⁎⁎	−0.002
	(0.373)	(0.332)	(0.371)	(0.014)	(0.006)
Caste OBC	0.505	0.563	0.506	0.058⁎⁎⁎	0.001
	(0.500)	(0.496)	(0.500)	(0.016)	(0.006)
Land farmed (Acres)	2.591	2.461	2.599	−0.130	0.008
	(3.971)	(3.000)	(3.726)	(0.102)	(0.060)
Sharecropping	0.308	0.331	0.295	0.023⁎	−0.014⁎⁎
	(0.462)	(0.471)	(0.456)	(0.013)	(0.007)

Observations	1,525	698	1,266		

Variables from detailed subsample:					
Primary school	0.643	0.641	0.681	−0.001	0.038⁎⁎⁎
	(0.480)	(0.480)	(0.467)	(0.002)	(0.010)
Secondary school	0.482	0.483	0.520	0.002	0.039⁎⁎⁎
	(0.500)	(0.500)	(0.500)	(0.003)	(0.010)
Asset index	0.129	0.121	0.258	−0.008	0.129⁎⁎⁎
	(1.605)	(1.598)	(1.607)	(0.010)	(0.031)
Permanent housing structure	0.550	0.547	0.581	−0.003	0.031⁎⁎⁎
	(0.498)	(0.498)	(0.494)	(0.003)	(0.010)
MNREGA assistance	0.263	0.259	0.246	−0.004	−0.017⁎⁎
	(0.441)	(0.439)	(0.431)	(0.003)	(0.008)
PDS assistance	0.646	0.645	0.626	−0.001	−0.019⁎⁎⁎
	(0.479)	(0.479)	(0.484)	(0.003)	(0.007)

Observations	711	698	594		

Notes: Household characteristics as reported in the midline survey by endline survey response status. This table restricts to those that reported growing pulses at midline and were therefore eligible for both endline survey modes. Top panel reports questions asked to all households; bottom panel reports questions asked to extended subsample. Columns 1–3 report sample mean and standard deviation; Columns 4–5 report difference in means from (1) and standard error of difference clustered at the village level.

*** Indicate significance at the 1 percent critical level.

** Indicate significance at the 5 percent critical level.

* Indicate significance at the 10 percent critical level.

While the demographic character of phone respondents is associated with greater agricultural output in general, sample selection alone cannot account for measured production gaps between survey modes. To quantify the importance of attrition, we take advantage of the 584 households that responded both in person and by phone, in 429 of which the same individual responded to both surveys. Self-reported production differences within these overlapping subsamples eliminate selection bias and isolate the direct effect of survey mode on the same household or individual responding to the same question over different media.^5^

**Fig. 2:**
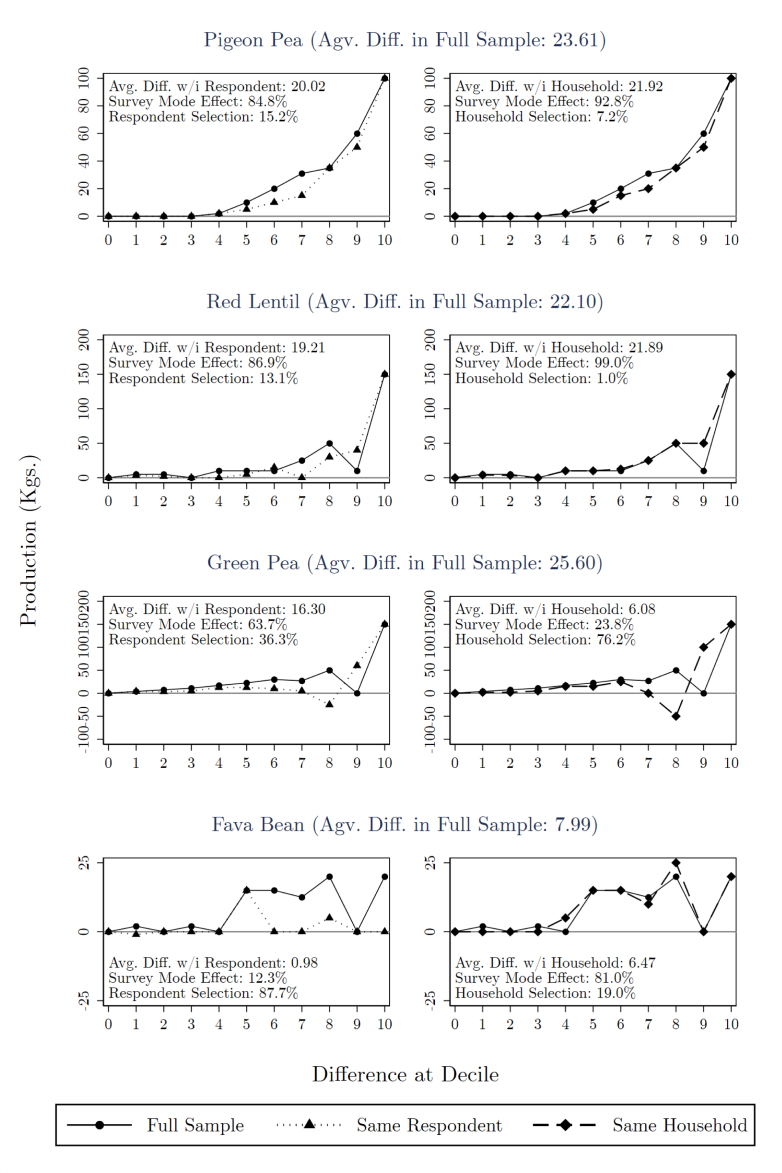
Difference at each decile in full and overlapping samples. Notes: Difference between self-reported production by phone and in person at each production decile in full and overlapping samples. Data for each crop includes only those who reported positive area for that crop at midline. Left column restricts to overlapping sample with same respondent; right column includes full set of overlapping households. Top production values are Winsorized to the 95th percentile independently by crop and by mode before computing mean difference.

We first discuss the effect of survey mode on responses given by the same individual. The left column of Fig. 2 compares differences between survey modes at each decile among those who responded to both surveys against differences across the full sample of respondents. The solid lines plot the production gap between survey modes at each decile in the full sample, reproducing results from Fig. 1, and reflect the net effect of both survey mode and differential selection. The dotted lines represent the production gap in the sample of overlapping respondents, which is only directly affected by survey mode.

For all four main pulse varieties, the production gap at each decile in the overlapping sample closely tracks that of the full sample. The largest deviations occur around the 60th to 80th percentiles of green peas and fava beans, and production reported in person actually exceeds that by phone at the 80th percentile for green peas. Other than this discrepancy, the gap between in-person and phone surveys that appears among the set of respondents who answered both surveys is of similar sign and magnitude to the difference in the full sample throughout the distribution of responses.

Comparing means across the subset of overlapping respondents confirms survey mode effects, rather than selective attrition, generate most of the measured production gap. For three out of four crops – pigeon pea, red lentil, and green pea – the within-respondent survey mode effect accounts for between 64% and 90% of the average difference across surveys. Only for fava beans is the average production gap within respondent less than half of that in the full sample.

Within-respondent differences reflect the pure effect of survey mode on the same individual answering the same question. This calculation eliminates heterogeneity caused by both selective attrition across households and by within-household selection of who responds. The latter channel, arising when different members participate in different survey types, can be considered part of the survey mode effect at the household level. Within-household survey mode effects, net of both the direct effect on respondents and household member selection, may be more informative for research design because attrition and the resulting bias can be measured, but researchers cannot know whether the same individual would have responded to different modes of contact.

Household-level survey mode effects explain an even greater portion of the production gap in general. The right column of Fig. 2 compares differences in self-reported production by survey mode in the full sample to differences in the sample of overlapping households, represented by the dashed line. The overlapping household sample, consisting of the 429 households in the overlapping respondent sample plus an additional 155 households in which different respondents answered each survey, tracks the full sample more closely across production deciles.

For the two main project crops – pigeon peas and red lentils – the shift from within-respondent to within-household comparisons increases the explanatory power of survey mode effects – from 85% to 93% and 87% to 99%, respectively. Moreover, for fava beans, the portion of the average production gap explained by household-level survey mode effects climbs to 81% with the addition of several households in which the primary farmers reports low production in person and another member reports higher production over the phone. Interestingly, we document a reversal for green peas as household-level comparisons introduce multiple cases in which the primary farmer reported substantially lower production over the phone than another respondent announced in person.^6^ While effects are not uniformly strong, these results taken together indicate most of the reported production difference between surveys does not come from differential attrition, but rather from the same respondent or household providing different answers based on the manner in which they were contacted.

### 3.2 Rounding and respondent engagement

We next consider differential respondent engagement by survey mode. Phone survey participants may be less engaged for a number of reasons—it is harder for remote enumerators to verify accuracy, it is easier to build rapport face-to-face, or it is more tempting to multitask while on the phone, to name a few. Low engagement would add measurement error to survey responses, and may bias responses upward in this context where production volumes are small to begin with.

As a proxy for respondent engagement, we present evidence of rounding in survey responses by plotting the frequency of each value for the right-most digit. Deviations from a smooth distribution, especially around numbers ending in zero and five, would indicate rounding. Gourlay et al. (2019) use crop cuts to show rounding frequently contributes to overestimation of self-reported production data.

**Fig. 3:**
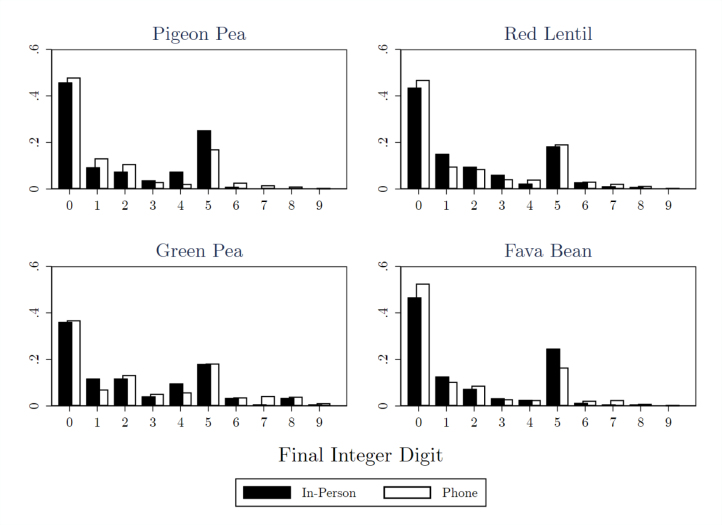
Right-most digit frequencies by survey mode. Notes: Fraction of non-zero responses with each value for rightmost digit by crop and by survey mode.

Rightmost-digit frequencies are plotted by survey mode and variety in Fig. 3. For each crop, we report the fraction of self-reported non-zero production values with each possible right-most digit by survey mode. The figure reveals an excess of responses that end in zero and five. Across all non-zero production data, these two last digits represent 64% percent of responses.

The fraction of responses ending in zero or five is consistent across survey modes. 44% of production values end in zero, 46% over the phone and 43% in person. Similarly, 19% of responses end in five, 18% over the phone and 21% in person. A chi-squared test fails to reject equality in rightmost-digit rounding at the 10% level. Moreover, the difference is so small that even if rounding caused respondents to double their self-reported production, it would only raise average production by 1% more by phone relative to in-person, well below the 14%–68% gaps reported in Fig. 1. These magnitudes imply that, while participants clearly round their responses, the influence of this behavior on differences by survey mode must be small.

Appendix A presents further evidence that respondent engagement does not appear to decay at differential rates between survey modes for the outcomes studied in this paper. However, we add the caveat that the pulse module was the first module asked in both surveys after consent and respondent identification, so it is unclear how well this finding would generalize over longer durations.

## 4 Treatment effect estimation

Results so far indicate population comparisons between surveys may be undermined by systematic differences caused by survey mode. In this section, we investigate how survey mode affects impact evaluation. This analysis is informative for researchers selecting a method of data collection or comparing results generated using different methods, for example when making inferences about how treatment effects evolve over time within a population.

For this analysis, we report impact evaluation results according to estimation of (1) separately by crop and survey mode. Estimation is straightforward for the in-person sample as it is drawn uniformly at random from the sampling frame. Production quantity is as reported for survey respondents with positive area planted and assumed to be zero for respondents with zero area planted. Regression following (1) produces a treatment effect estimate inclusive of attrition bias caused by survey non-response.

Comparable estimation in the phone sample is confounded by the fact that enumerators did not attempt to contact households with zero area devoted to pulses at midline. Therefore, the sample consists of a subset of households – those with positive area planted – subject to the attrition pressures induced by phone surveying and a complementary subset – those with no area planted – with known production volume but an unknown phone response rate. These groups are endogenously determined because area planted at midline may be affected by treatment.

To estimate the effect of treatment in the phone sample, we run a weighted least squares regression following (1). Households that responded to the phone survey are assigned a weight of 1, and households with zero area planted are assigned a weight of 0.83 corresponding to the response frequency among surveyed households. Because all non-planting households have an identical production value of zero, this regression recovers the estimated treatment effect inclusive of phone-induced attrition bias under the assumption that phone response rates among non-planting households would have been comparable to response rates among planting households.

**Fig. 4:**
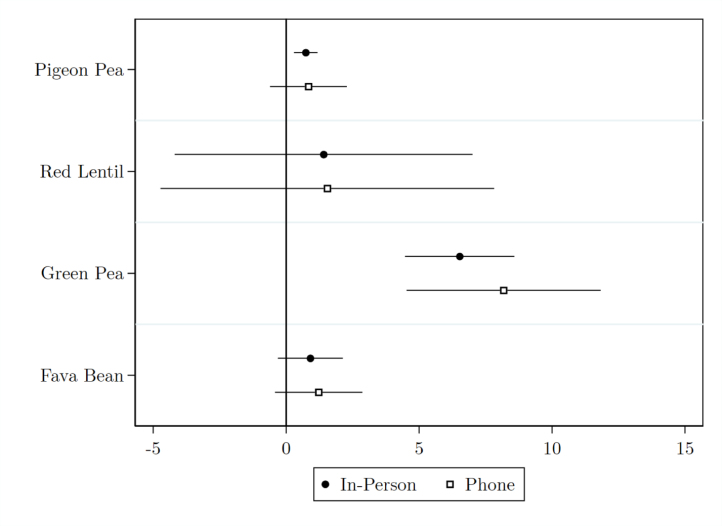
Treatment effect estimates by survey mode. Notes: Coefficient estimates for treatment effect according to (1) by crop and by survey mode. Error bars represent true 95% confidence intervals for estimation using phone survey data. For estimation using in-person survey data, 95% confidence intervals are shrunk by $\sqrt{N^{InPerson}/N^{Phone}}$ to represent the hypothetical confidence interval had the in-person survey had the same number of respondents as the phone survey. Top production values are Winsorized to the 95th percentile independently by crop and by mode before regression estimation.

Regression coefficients are presented in Fig. 4 with 95% confidence intervals subject to a standard error adjustment for sample size. In general, regression standard errors are computed as

(2) $$\sigma_{\beta }=\frac{\sigma_{\epsilon }}{\sqrt{N}}$$

a ratio of the residual variance and the sample size, both of which vary by survey mode in our data. In Fig. 4, we isolate the residual variance component of (2) by multiplying $\sigma_{\beta }^{InPerson}$ by $\sqrt{N^{InPerson}/N^{Phone}}$. This correction approximates the regression standard error we would have computed had the in-person survey reached as many respondents as the phone survey while maintaining the same residual variance.^7^

Estimated treatment effects are nearly identical in magnitude across survey modes for all four main pulse crops, and a standard t-test fails to reject equality for any crop. This fact remains true even after the $\sqrt{N}$ standard error correction described above, which shrinks the in-person standard errors and thereby raises the probability of rejection. Exact coefficients and standard errors are reported in Appendix B. Notably, the higher attrition rates among pulse producers in the phone survey do not appear to introduce bias. These results indicate that, in contrast to the findings on population moments in the previous section, treatment effect estimation remains stable across survey modes. That is, any systematic differences between in-person and phone responses appear consistently in both treatment and control.^8^

While regression coefficients remain stable, Fig. 4 shows that standard errors are consistently smaller in the in-person data. This discrepancy highlights a tradeoff in study design: phone surveys, while usually cheaper, generate noisier data. The standard error approximation in (2) provides a straightforward quantification of this tradeoff. To estimate the effect of treatment on pigeon pea production with equal precision, the phone survey would have needed to be 10.7 times larger than the in-person survey; 1.2 times for lentils; 3.1 times for green peas; and 1.8 times larger for fava beans. That is, the cost per response may need to be up to 10.7 times lower over the phone than in person, depending on the outcome of interest, for phone surveying to be a cost-effective method to improve study power.

Estimates in Fig. 4 control for household fixed characteristics elicited in person at midline. Dropping these covariates lowers precision, but point estimates remain stable and the relative difference in standard errors persists. This same pattern of consistent point estimates but larger standard errors in phone survey data also appears when restricting to the overlapping subsample^9^ of households that participated in both surveys. The implied cost ratio in these specifications leans slightly more in favor of in-person surveying.

## 5 Conclusion

Overall, this study uncovers meaningful differences in the sample distribution of agricultural output across survey modes. We show a systematic pattern of higher self-reported production over the phone relative to in person, even within the same respondent, which may bias estimates of local or regional productivity. It remains an open question which mode more closely approximates the truth. Validating survey-based production measures would require more resource-intensive methods such as sub-plot crop cuts or monitoring of full plot harvests (see Lobell et al., 2020, Kosmowski et al., 2021), and such validation is beyond the scope of this study.

The discrepancy between survey modes is consistent with greater social desirability bias among phone respondents. Surveys were part of an initiative to increase pulse production, a goal well understood by both treatment and control farmers, and the survey mode effect is most apparent among the two crops explicitly promoted by the intervention. Evidence of social desirability bias among phone survey respondents, possibly related to enumerators’ inability to verify responses, has previously been found in studies of agricultural productivity (Kilic et al., 2021), student performance (Crawfurd et al., 2021), political attitudes (Holbrook et al., 2003), and urban microentrepreneurship (Garlick et al., 2020). The former three settings produce similar evidence that phone surveys generate more socially desirable population outcomes. Among urban microentrepreneurs, this bias manifests in self-reported data reliability – whether respondents claim to keep written records – but not in business outcomes, which may be equally difficult to verify by phone and in person.

Our findings more generally highlight a potential challenge in maintaining long-term databases such as those produced by national statistical offices. Time-series population statistics may be disrupted as survey units update procedures to take advantage of more pervasive information and communication technologies. Improved aggregation and imputation methods have already proven to generate discontinuities in historical trends (Jerven, 2013). Survey-mode-induced disruptions may be more difficult to detect because they coincide with technological expansions that cause real deviations from trend, and will be especially obscured where new survey methods were adopted out of necessity during the COVID-19 pandemic. In such cases it will be imperative to design surveys that allow researchers to reconcile new and old data, and eliminate artifacts of the method of data collection.

Somewhat reassuringly, survey mode effects appear to be less concerning for bias in program evaluation. Gaps in self-reported agricultural production are consistent across experimental study arms and therefore do not influence the magnitude of estimated program impacts. Data differences by survey mode are nevertheless important for research design due to precision. We report higher sampling variation in outcome data by phone, though the influence of this difference varies by outcome. In-person surveying at midline further improved precision by allowing us to control for household characteristics. If these covariates were measured more poorly or not at all by phone at midline, the gap in precision between survey modes would have been even greater. Overall, our results caution phone surveying may not save on costs if larger sample sizes are needed to achieve the same level of power.

Implementation experience raises two additional research design considerations not directly quantified in this analysis. First, different survey modes may have different levels of success in reaching specific household members for participation. In our study, in-person enumerators reached the primary farmer slightly more frequently than phone surveyors. Relative success rates may vary across different contexts.

Second, while we focus on the subset of outcomes elicited both in person and by phone, surveys also varied in the scope of their questionnaires. Specifically, enumerators were able to spend over an order of magnitude more time with respondents in person. As a result, in-person surveys generated substantially more data, including production volume for a wider range of crops as well as detailed modules on household income, consumption, and food storage. The ability to reach desired respondents and the breadth of data per respondent add additional dimensions to the tradeoff between cost and precision when selecting a mode of survey for program evaluation.

## Declaration of competing interest

The authors declare that they have no known competing financial interests or personal relationships that could have appeared to influence the work reported in this paper.

## Data Availability

Data will be made available on request.

## Appendix A Supplementary data

MMC S1 Supplementary details on participation and timing, respondent fatigue, responses by quantile, and regression results.

## Footnotes

1 A complementary application of mobile phone data avoids surveying altogether and draws inferences about household-level outcomes from metadata (see Blumenstock et al., 2015).

2 Biemer, 2001, Nord and Hopwood, 2007 analyze panel data from national statistical offices where the first survey round is conducted in person and subsequent rounds by phone, but this structure does not allow separate identification of survey mode and time effects within household.

3 https://www.3ieimpact.org/evidence-hub/publications/systematic-reviews.

4 https://www.cochranelibrary.com/cdsr/reviews.

5 In Appendix A we show results are not influenced by differences in survey timing.

6 The full breakdown of within-household and within-respondent differences are presented in Appendix B.

7 $N^{Phone}$ is calculated as the sum of regression weights. Note this is a simplified approximation because the regression standard errors are clustered at the village level, which is the unit of random assignment to treatment. A more comprehensive correction would need to fully specify differences in the number of clusters, observations per cluster, and intra-cluster correlation by survey mode.

8 Survey mode may still play a role in the interpretation of outcomes if treatment effects are benchmarked against the control mean or reported in standardized units.

9 The in-person sampling frame included households that reported zero area planted at midline, but these households were excluded from phone surveying. To recover consistent treatment effects for this exercise, we supplement the overlapping sample with all zero-area households that were selected for in-person surveying, weighted by the phone-survey attrition rate as before.
