# Supplementary material for: Does survey mode matter? Comparing in-person and phone agricultural surveys in India
Source: J Dev Econ. 2024 Jan;166:103199. doi: 10.1016/j.jdeveco.2023.103199 (PMC10729321; doi:10.1016/j.jdeveco.2023.103199)
Supplement: MMC S1 — Supplementary details on participation and timing, respondent fatigue, responses by quantile, and regression results. [file mmc1.pdf]

Supplementary Appendix for

"Does Survey Mode Matter?"

For Online Publication Only

A Survey Details

A.1 Sample Selection and Response Rates

The impact evaluation took place among 6,971 households randomly assigned to treatment and control. Out of the evaluation population 2,346 households were selected at random for in-person midline surveying. This set of midline respondents constitutes the sampling frame for the current study, and households' midline survey status determines their first-year endline survey method.

Among midline survey respondents, 1,100 were selected at random for an extended questionnaire on socioeconomic status. Researchers attempted to contact all of these households again in person at the first-year endline, and successfully surveyed 1,055 of them. This subset, drawn at random from the sampling frame, constitutes the in-person sample in our analysis.

At midline, 1,525 respondents reported some positive area devoted to pulse cropping. Researchers attempted to contact all of these households by phone at the first-year endline, and successfully surveyed 1,266 of them. All midline survey respondents owned a mobile phone, so non-response was caused by either not answering the call or declining to participate in the survey. This non-random subset of the sampling frame constitutes the phone sample in our analysis.

711 households both reported positive pulse area at midline and were randomly selected for extended surveying. As a result, researchers attempted to contact these households both in-person and by phone, and successfully reached 584 of them through both modes. This subset of the sampling frame constitutes the overlapping sample in our analysis. A complete breakdown of midline survey status and endline survey response is presented in Table S1.

Aggregate data are not directly comparable between the in-person and phone samples because the

Table S1: Midline Survey Status and Endline Survey Response

Midline status:			Pulse Area (Phone Sample)		
			Positive		Zero
		Endline:	Responded	No Response	Not Contacted
Extended Surveying (In-Person Sample)	Yes	Responded	584	114	357
		No Response	10	3	32
	No	Not Contacted	672	142	432

Notes: Number of households contacted and number of respondents for each survey mode. The full sampling frame consists of all 2,346 households selected at random from the study population for midline surveying. Households randomly selected for extended surveying at midline were contacted for in-person surveying. Households reporting positive pulse area at midline were contacted for phone surveying.

samples were selected differently. In particular, the phone sample screens for positive pulse area at midline, which is straightforwardly correlated with production at endline. We address this fact in two ways. All analysis in Section 3 on self-reported production restricts the in-person sample to the phone-eligible subset of respondents. This group represents a random sampling of those contacted by phone. Section 4 on treatment effects cannot use the same restriction because pulse area is an outcome of treatment. Analysis in this section instead reweights data in the phone sample to simulate phone-based attrition rates among those with zero pulse area at midline.

A.2 Days Elapsed between Surveys

Surveys were carried out separately by different research teams, so among the overlapping sample of respondents there is incidental variation in which survey was conducted first and in the time elapsed between surveys. The phone survey was conducted an average of one week prior to the in-person survey, but timing differences vary from the phone survey being conducted 26 days before the in-person survey to 13 days after. The full distribution of the gap between surveys in the overlapping sample is shown in Figure S1.

Survey timing does not seem to affect self-reported production volume. In Figure S2 we plot the production gap between phone and in-person survey responses against the days elapsed between surveys by household for each of the four major crops. In all four cases, there is little relationship between differences

Figure S1: Days Elapsed from Phone Survey to In-Person Survey

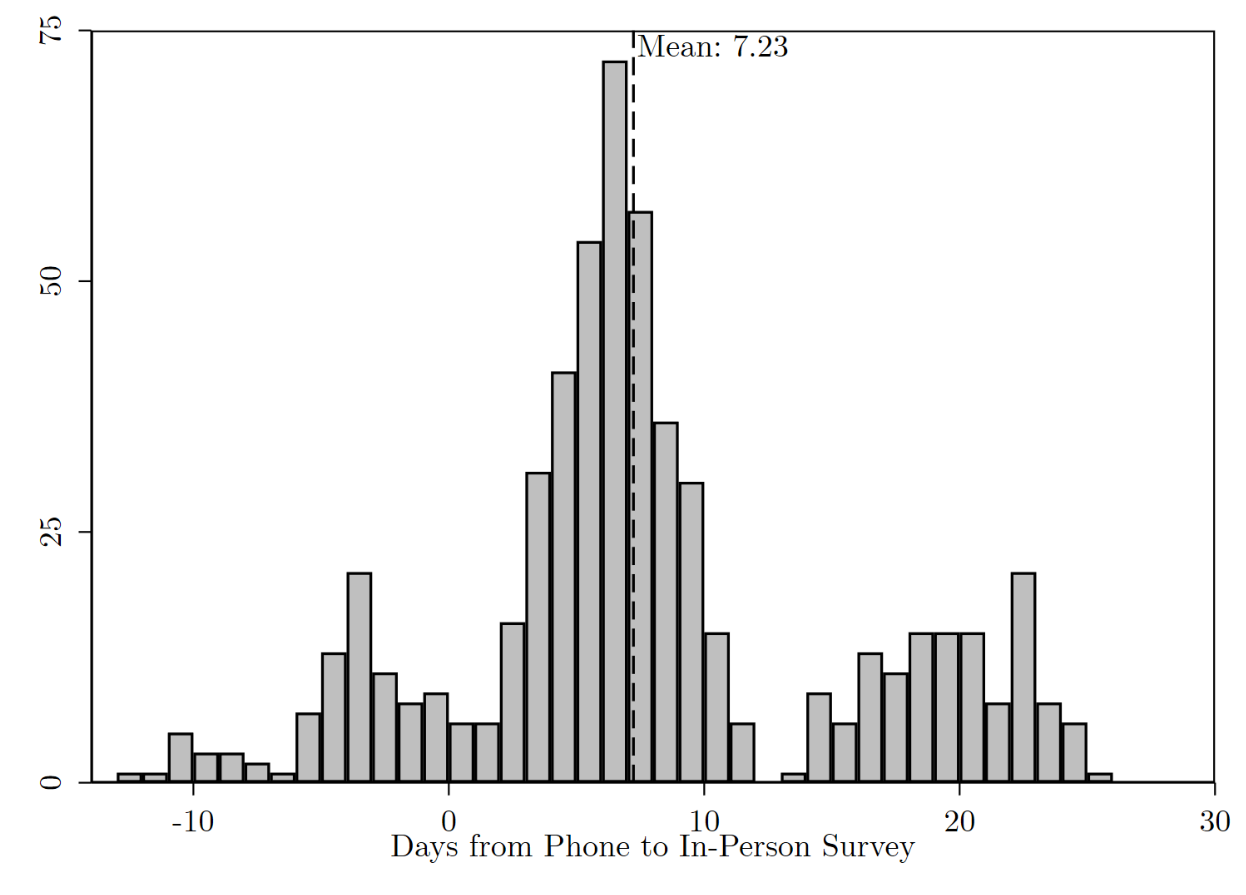

Notes: Histogram of days between phone and in-person surveying among households that participated in both surveys. Negative values indicate the in-person survey took place before the phone survey.

in timing and differences in self-reported production, indicating our results are not an artifact of incidental differences in the exact day when respondents were surveyed.

Figure S2: Production Gap by Difference in Survey Timing

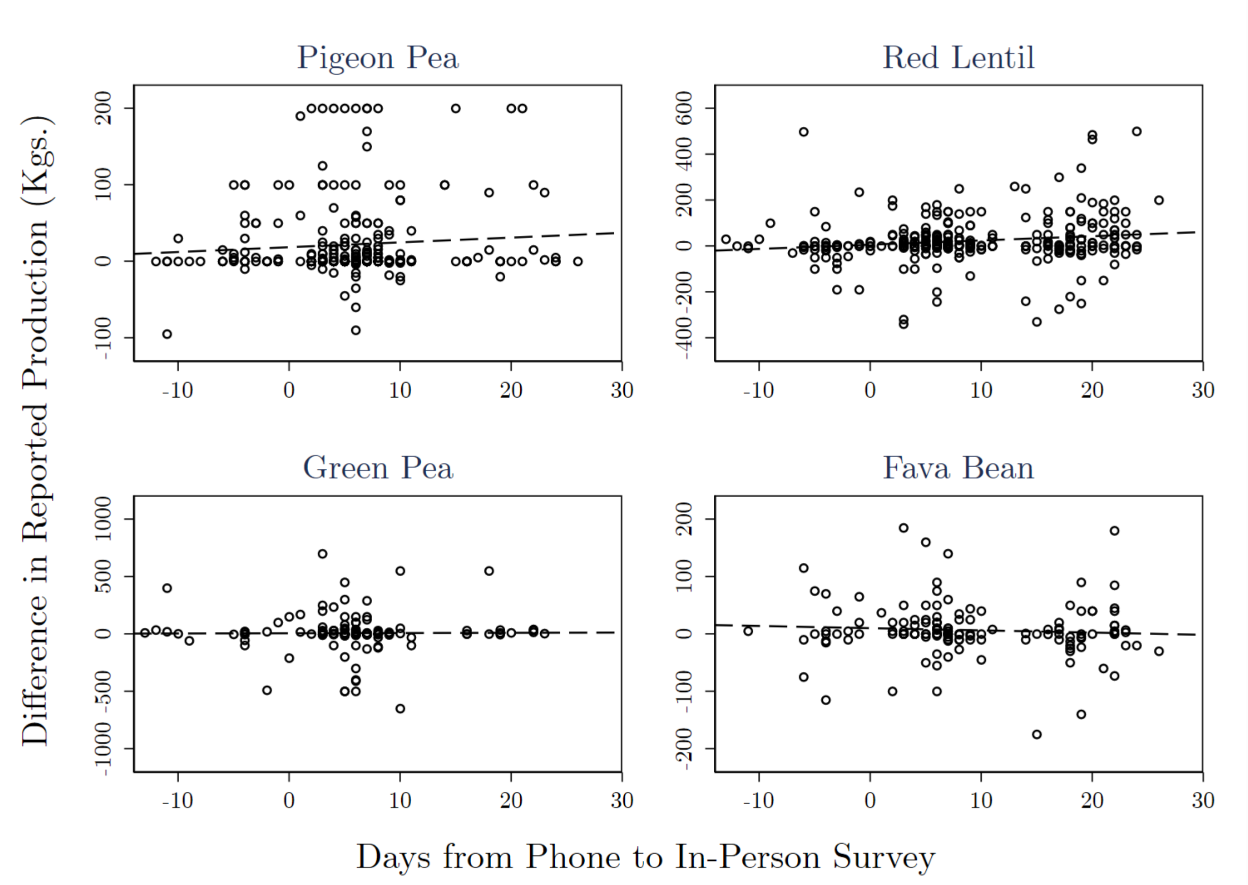

Notes: Data from households that participated in both surveys only. X-axis represents days elapsed between phone survey and in-person survey. Y-axis represents self-reported production by phone minus self-reported production in person. Dashed line shows best linear fit.

Table S2: Relationship between Survey Duration and Rounding

	Questions Prior		Words Prior	
	In-Person	Phone	In-Person	Phone
Pigeon Pea	-0.2006	-0.0019	-0.0306	0.0000
	(0.0132)	(0.0186)	(0.0013)	(0.0047)
	Δ = 0.02		Δ = -1.08	
Red Lentil	-0.0009	0.0042	-0.0001	0.0002
	(0.0027)	(0.0028)	(0.0003)	(0.0002)
	Δ = 0.00		Δ = 0.01	
Green Pea	-0.0017	-0.0052	-0.0001	-0.0005
	(0.0053)	(0.0054)	(0.0006)	(0.0003)
	Δ = -0.00		Δ = -0.01	
Fava Bean	-0.0073	0.0027	-0.0006	0.0001
	(0.0025)	(0.0023)	(0.0003)	(0.0002)
	Δ = 0.04		Δ = 0.07	

Notes: Each cell reports the coefficient from a regression of the frequency of rounding on survey duration by crop, survey mode, and duration measure with standard errors clustered at the household level in parentheses. The third row in each cell reports the predicted difference in rounding frequency between survey modes, calculated as difference in regression coefficients multiplied by the difference in average duration.

A.3 Survey Duration and Respondent Fatigue

The pulse module was the first module, so the timing of questions within each survey mode is comparable. To test for differential fatigue by survey mode, we evaluate both the frequency of rounding and the coefficient of variation in responses as a function of the number of questions and the number of words that came prior in the survey, and we find no evidence of differential decay in quality by survey mode. Table S2 reports results from regressions of the frequency of rounding on number of prior words and questions by crop and by mode, and Figure S3 plots the coefficient of variation among non-zero responses by these two measures. Ideally we would have extended this analysis to include survey time elapsed, but this outcome was unfortunately not recorded by our survey software.

Differences in response quality predicted by survey duration between survey mode are quantitatively small. The third row of each cell in Table S2 reports the predicted difference in rounding frequency between survey modes, calculated as difference in regression coefficient between modes multiplied by the average

Figure S3: Relationship between Survey Duration and Coefficient of Variation

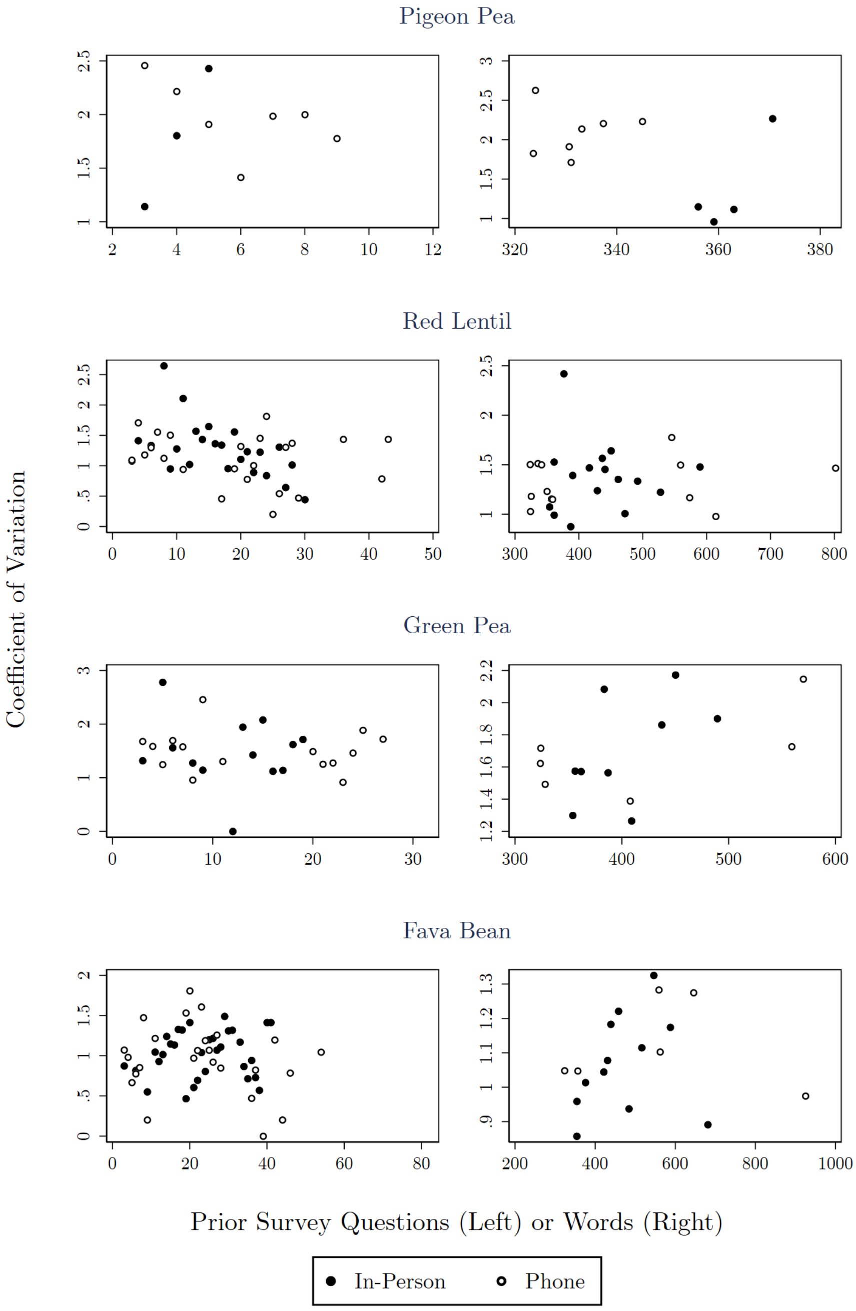

Notes: Coefficient of variation among non-zero survey responses by survey duration. Coefficient of variation is computed as the standard deviation among responses divided by the mean. Each point represents coefficient of variation within a bin of roughly 25 responses, and the x-axis shows the mean survey duration within the bin.

difference in survey duration before the reported crop. These predicted differences are almost all small in magnitude, with the only exception being number of words before asking about pigeon pea production. This estimate comes about because each survey had slightly different introductory text, and pigeon pea was the first crop we asked about, so the small difference in coefficients between surveys is magnified by a large gap in prior survey words.[10] Figure S3 visually confirms this pattern of little difference in precision between surveys, but a large gap in the support of number of words said before asking about pigeon peas. Note that this analysis is suggestive but not causally identified because survey duration is endogenous to farmers' choice of diversity in crops planted.

[10]The difference estimate for words prior to pigeon pea production actually predicts that the phone survey would have substantially less rounding than the in-person survey.

A.4 Survey Questionnaires

Questions on crop production were harmonized between in-person and phone surveys. Excerpts containing the exact wording delivered to enumerators for translation into the local language is given below. We show the in-person questionnaire first followed by the phone questionnaire.

Crop name {cropname} will be preloaded from existing data. The number of plots that each crop was cultivated on in Rabi {cropplots} will also be preloaded.

First, I would like to confirm that we have correct information on the crops you cultivated during the last Rabi season.<br>**Ask for each crop that the farmer reported cultivating in the 2017-2018 Rabi season (except orchard crops).**		
B1	You reported cultivating {cropname} during the last Rabi season. Do you confirm that you cultivated {cropname}?<br>Hint: You reported cultivating {cropname} on a total of {cropplots} plots. This includes any {cropname} that you cultivated, for household consumption or commercial purposes, and includes any {cropname} cultivated on the border of your plots.	1. Yes<br>2. No – Skip to Next Crop

Module C: Production outputs and revenues (Pigeon pea + Rabi crops)

Ask for each crop that the farmer reported cultivating in the 2017-2018 Rabi season (except orchards and any crop that the farmer reported as not cultivating in Module B).

Now we are going to ask some questions about your harvest of {rabi_crop) from all plots.		
C1	How much {rabi_crop} was harvested from all plots in the recent Rabi harvest?<br>Hint: Please enter 0 if {rabi_crop} has not yet been harvested or has only been partially harvested.	
(a)	Quantity:<br>Hint: Please enter 0 if {rabi_crop} has not yet been harvested. Enter -999 if respondent does not know and -998 if respondent refuses to answer.	
(b)	Unit:	1. KGs<br>2. Quintals<br>3. Grams<br>4. Liters<br>5. Passeri<br>6. Mann<br>7. Piece
C2	If the farmer answered 0: Why did you not harvest any {rabi_crop}?	1. Could not afford to cultivate the whole season<br>2. Insects<br>3. Rodents/pests<br>4. Flood<br>5. Theft<br>6. Harvest period is later<br>7. Still harvesting

Section 2: Kharif/Rabi Production				
First, I would like to confirm that we have correct information on the pulses you cultivated during the past Kharif and Rabi season. Then I will ask some questions about your harvest of pulses from all plots.<br>**Note: Kharif was roughly June-November 2017 and Rabi was November 2017 - April 2018**				
**Repeat 2.1-2.12 for each pulse that the farmer reported cultivating.**				
2.1	Did you harvest {crop} during this past Kharif or Rabi season?	0. No		
		1. Yes		Skip to 2.4
2.2	Did you grow {crop} in the 2017 Kharif or 2017-2018 Rabi season?<br>When our surveyor came to your household, they asked you about the crops you grew on your agricultural plots. Previously you identified {crop} on the following plots: {landmark1}, {landmark2}, {landmark3}, ...	0. No, did not grow		Return to 2.1 for next crop
		1. Yes, grew crop and harvested		Skip to 2.4
		2. Yes, grew crop but did not harvest		
2.3	Why did you not harvest any {crop}?	1. Could not afford to		
		2. Insects		
		3. Rodents/Pests		
		4. Flood		
		5. Theft		
		6. Harvest period is later		
2.8	How much {crop} was harvested from all plots in the most recent harvest?	Quantity:	1. kg	Skip to 2.10
			2. quintal	
			3. gram	
			4. paseri	
			5. maund	
			6. bags	

B Quantitative Results

B.1 Tests of Equality at Quantiles

Comparisons of the fraction of farmers reporting non-zero production and fractions above the median, 75^{th}percentile, and 90^{th}percentile by survey mode for each of the four main crops are presented in Table S3. The third column presents p-values from a ξ^2 test for equality between surveys. To test equality at each percentile, we first calculate the grand value at that percentile across both samples. Then, we test for equality in the fraction of respondents in each survey that report production that exceeds the grand percentile value.

Note that in a few cases, fewer than $100 - N$ percent of respondents report production above the grand N^{th}percentile in both surveys. These cases correspond to situations where many responses are bunched exactly at the value at that percentile. We compare the fraction in each sample that report strictly greater production, excluding all those bunched at that percentile value. Inversely, had we compared fractions reporting greater-than-or-equal-to that level of production, such bunching would have generated cases where more than $100 - N$ percent of respondents were counted above the N^{th}percentile in both surveys.

B.2 Within-Household and within-Respondent Differences

Figure S4 plots self-reported production over the phone against self-reported production in person by crop for households that responded to both surveys, with shaded dots denoting households in which the same individual answered both surveys. Figure S5 plots the difference between phone and in-person responses by crop. Each panel shows the correlation of the within-household discrepancy across crops.

B.3 Program Evaluation Regression Results

Table S4 reports regression results from (1) corresponding to the estimates plotted in Figure 4. Control variables X_i include block fixed effects and respondent age, gender, caste, and experience growing pulses in prior years, and standard errors are clustered at the village level. Standard errors in Table S4 are not adjusted for sample size differences according to (2).

Table S3: Fraction at Various Percentiles by Survey Mode

	Fraction of Respondents		χ^2 Test
	In-Person	Phone	p-value
Pigeon Pea Production:			
Greater than Zero	0.34	0.64	0.00
Above Median	0.32	0.59	0.00
Above 75th Percentile	0.11	0.31	0.00
Above 90th Percentile	0.00	0.09	0.00
Red Lentil Production:			
Greater than Zero	0.89	0.94	0.00
Above Median	0.45	0.51	0.04
Above 75th Percentile	0.19	0.25	0.03
Above 90th Percentile	0.09	0.10	0.50
Green Pea Production:			
Greater than Zero	0.83	0.95	0.00
Above Median	0.42	0.50	0.08
Above 75th Percentile	0.20	0.24	0.31
Above 90th Percentile	0.08	0.10	0.57
Fava Bean Production:			
Greater than Zero	0.90	0.91	0.64
Above Median	0.43	0.52	0.05
Above 75th Percentile	0.18	0.25	0.08
Above 90th Percentile	0.08	0.09	0.69

Notes: The first two columns report the fraction in each survey reporting non-zero production values and fraction in each survey reporting production strictly greater than the 50th, 75th, and 90thgrand percentiles across both surveys by crop. The final column reports p-values from a χ^2 test of equal fractions between surveys.

Figure S4: In-Person and Phone Responses by Crop in Overlapping Household Sample

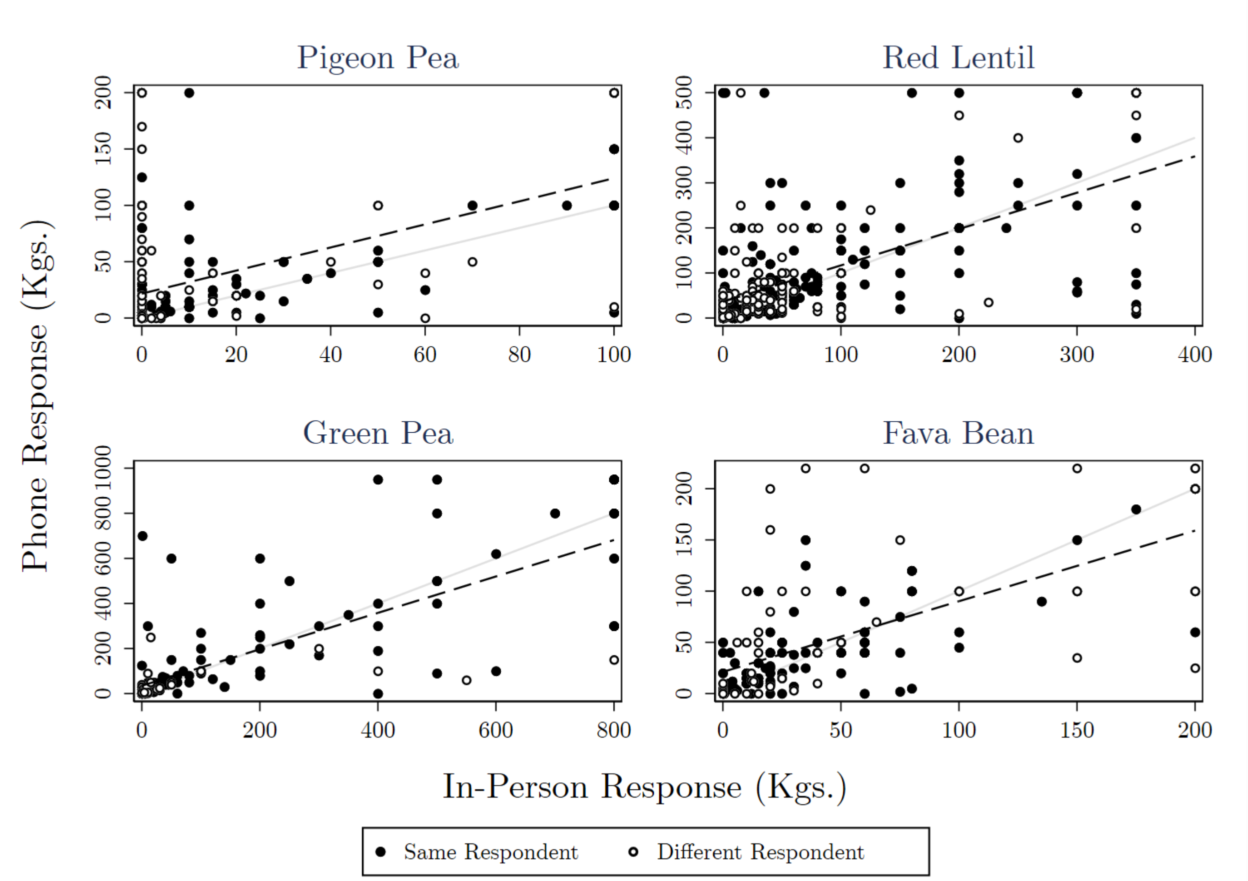

Notes: Production reported by phone plotted against production reported in person by crop among households that answered both surveys. Filled dots represent same respondent; hollow dots represent households with different respondent for each survey. Dashed lines represent best linear fit. Gray lines represent 45-degree line on each graph.

Figure S5: Within-Household Difference by Crop in Overlapping Household Sample

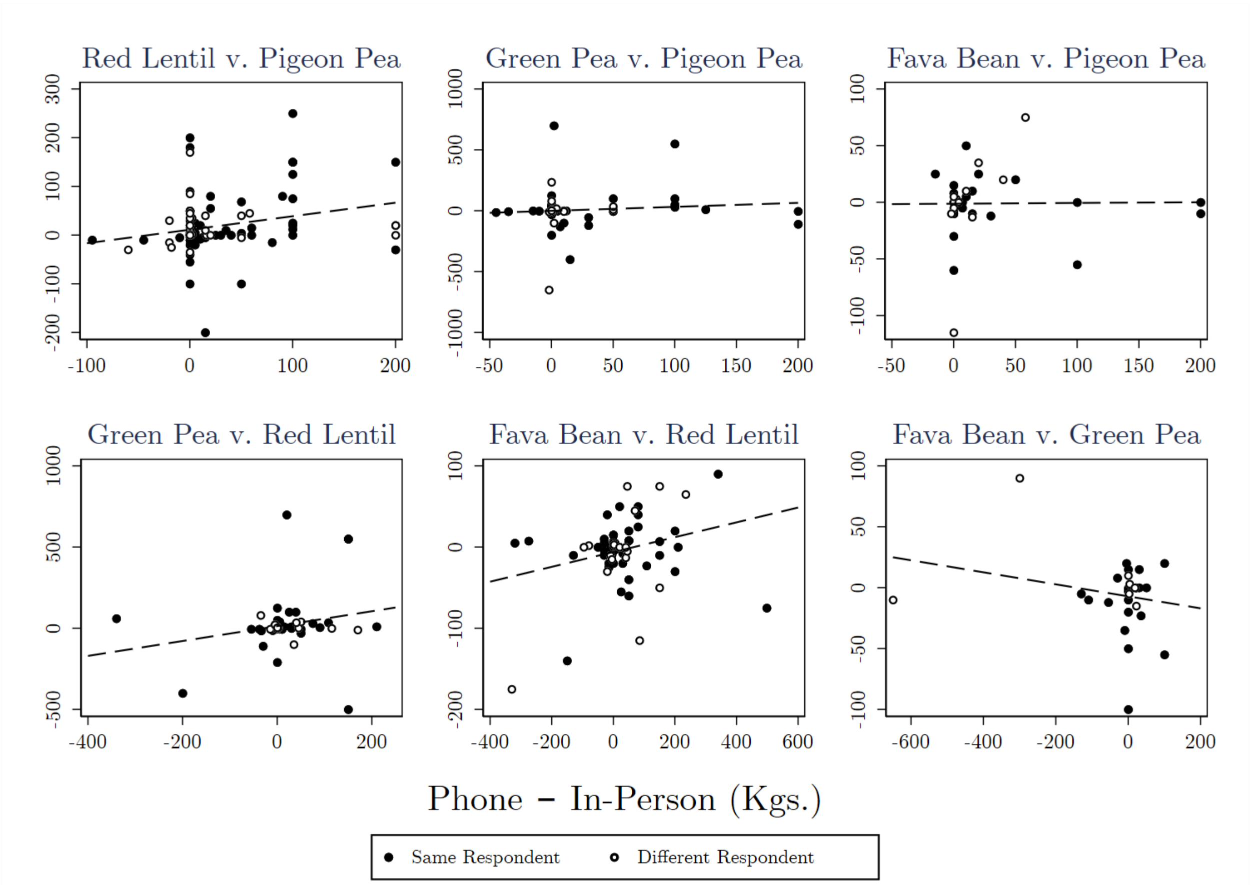

Notes: Difference in production reported by phone and in person by crop among households that answered both surveys and grew multiple crops. Graph title denotes y-axis v. x-axis. Filled dots represent same respondent; hollow dots represent households with different respondent for each survey. Dashed lines represent best linear fit.

Table S4: Treatment Effect Estimates: Regression Results

	Pigeon Pea		Red Lentil	
	In-Person	Phone	In-Person	Phone
Treated	0.737	0.840	1.410	1.549
	(0.325)	(0.732)	(4.122)	(3.178)
Control Mean	0.72	4.09	25.16	25.75
R-Squared	0.11	0.11	0.16	0.18
Observations	1055	2079	1055	2079
	Green Pea		Fava Bean	
	In-Person	Phone	In-Person	Phone
Treated	6.526	8.179	0.910	1.222
	(1.514)	(1.849)	(0.900)	(0.833)
Control Mean	2.48	4.11	4.13	4.91
R-Squared	0.13	0.14	0.08	0.09
Observations	1055	2079	1055	2079

Notes: Estimated treatment effect by survey mode following (1). Every specification includes block fixed effects and controls for respondent age, gender, caste, and experience growing pulses in prior years. Standard errors clustered at the village level reported in parentheses. ***, **, and * indicate significance at the 1, 5, and 10 percent critical level.
